# AIMing 2 promote lupus by targeting helpers

**DOI:** 10.1002/ctm2.844

**Published:** 2022-05-11

**Authors:** Jianmei W. Leavenworth

**Affiliations:** ^1^ Department of Neurosurgery University of Alabama at Birmingham Birmingham Alabama USA; ^2^ Department of Microbiology University of Alabama at Birmingham Birmingham Alabama USA; ^3^ The O'Neal Comprehensive Cancer Center University of Alabama at Birmingham Birmingham Alabama USA

1

The immune system defends the body against foreign or dangerous invaders while protecting the self. If it mistakes self for non‐self, then it attacks the body's own tissues, causing autoimmune disorders, such as systemic lupus erythematosus (SLE). SLE is the most common type of lupus. Many organs, including the skin, joint, lung, kidney and brain, can be affected and manifested with widespread inflammation and tissue damage. The accumulation of a wide spectrum of autoantibodies against self‐nuclear components is a hallmark of SLE.^1^ Follicular helper T (T_FH_) cells, a subset of CD4^+^ T helper (T_H_) cells, are required for the precise control of antibody production by germinal centre (GC) B cells.^2^, ^3^ It is well‐recognised that over‐expansion or dysregulation of T_FH_ cells is the root of the aberrant production of self‐reactive antibodies that promote lupus pathogenesis.^4^ Thus, a deeper understanding of the mechanisms controlling T_FH_ cell differentiation and function may facilitate the development of therapies to treat SLE.

Wu et al. have presented a new mechanism by which the T_FH_ response is potentially dysregulated in SLE patients. The expression of Absent in melanoma 2 (human AIM2 and murine Aim2) is found to be increased in T_FH_‐like cells (CD4^+^Bcl6^+^PD1^+^) in the peripheral blood and skin lesions of lupus patients, compared to healthy controls. The elevated AIM2 level is attributed to the increased recruitment of the hydroxymethyltransferase ten‐eleven translocation 2 (TET2) driven by the cytokine interleukin (IL)‐21 to the *AIM2* promoter region, leading to reduced *AIM2* methylation. AIM2 interacts with the T_FH_‐promoting transcription factor c‐MAF,^3^ enhancing and sustaining IL‐21 production and T_FH_ cell differentiation. Accordingly, deletion of Aim2 or Tet2 in murine T cells results in reduced T_FH_ and GC B cells as well as antigen‐specific antibody production. Serum autoantibody levels and renal immunoglobulin G deposition are also expectedly reduced in mice with T‐cell‐specific ablation of Aim2 in both pristane‐induced and chronic graft‐versus‐host disease lupus models. The increased expression of *IL21*, *TET2*, *AIM2* and *c‐MAF* and the positive correlation of *AIM2* with *IL21* or *c‐MAF* in SLE patients support the potential contribution of the IL‐21‐TET2‐AIM2‐c‐MAF axis to lupus development. Nevertheless, TET2, as a DNA demethylating factor, has various target genes. In fact, IL‐21 increases the recruitment of TET2 to the *BCL6* promoter region in lupus patients, while interferon (IFN)‐α, the signature cytokine for lupus, also increases *AIM2* levels, albeit to a lesser degree. These results imply that multiple pathways promote T_FH_ dysregulation in SLE.

AIM2, a family member of the pyrin and HIN domain‐containing proteins, is a sensor for cytosolic DNA, which plays a crucial role in the regulation of the inflammasome and innate immune response.^5^ The inflammasome‐independent function of AIM2 is emerging, as it has been investigated in cells belonging to the adaptive immunity, such as B cells, regulatory T (Treg) cells and also T_FH_ cells reported by Wu et al.^6^, ^7^ The mechanisms of action vary among these cell types, ranging from immune metabolism alterations to transcriptional regulation. The distribution of AIM2 within the cell may dictate its function, as AIM2 mainly resides in the nucleus of CD4^+^ T cells, which may aid in its regulation of the c‐MAF‐dependent transcriptional program to promote T_FH_ cell differentiation.

AIM2 is expressed at the highest levels in T_FH_ cells compared to other T_H_ cells. Further analysis of mice with a T‐cell‐specific deletion of Aim2 may allow us to precisely determine whether Aim2 regulates the differentiation of other T_H_ cells, particularly T_H_17 cells, as IL‐21 and c‐Maf also regulate T_H_17 development.^8^ Since controlling the AIM2 expression level is the key to T_FH_ cell fate determination, it is also important to understand why AIM2 levels decline gradually during in vitro T_FH_ cell differentiation. Elucidating factors that modulate AIM2 expression in T_FH_ cells may guide the strategies to target T_FH_ cells in SLE and other autoimmune disorders.

Besides T_H_ cells, Treg cells express Aim2, and Aim2 is important for the maintenance of stable suppressive Treg cells in a murine experimental autoimmune encephalomyelitis model.^7^ Follicular regulatory T (T_FR_) cells, a subset of Treg cells, share many features with T_FH_ cells but play an important role in restraining T_FH_ and GC B‐cell responses to prevent autoantibody production.^2^, ^9^ Targeting T_FH_ cells to treat lupus or other autoimmune disorders needs to consider keeping T_FR_ cells intact. In this regard, understanding whether AIM2 regulates the differentiation and function of T_FR_ cells differently from T_FH_ cells will be important.

The same research team also recently revealed higher AIM2 expression in memory B cells and plasma cells from the circulation and skin lesions of lupus patients.^6^ IL‐10 has a greater capacity than IL‐21 to upregulate AIM2 in B cells by reducing the DNA methylation level of *AIM2*.^6^ Importantly, conditional knockout of Aim2 in B cells attenuates lupus symptoms and reduces T_FH_, GC B cells and plasma cells in the pristane‐induced lupus model.^6^ It is quite fascinating that both T_FH_ cells and B cells adapt to the lupus environment by employing epigenetic regulation via upregulating AIM2, despite their responses to different triggers. Given the specific impact of autoantibody isotype, specificity or affinity to self‐antigens on SLE pathogenesis,^1^ it is imperative to evaluate whether AIM2 expression in T_FH_ cells or B cells regulates the antibody class switching and/or affinity maturation.

Currently, there is no cure for lupus. Therapies targeting B cells, the autoantibody producer, have shown some promise,^10^ but targeting B cells may not be sufficient, given the far more complicated regulation of B cells, including the newly identified pathways emanating from T_FH_ cells. The increased AIM2 in T_FH_ cells of SLE patients but not in psoriasis or healthy people suggests its potential as a diagnostic marker for SLE. However, the involvement of AIM2 in the regulation of multiple cell types suggests that it may not be an ideal therapeutic target. Moreover, epigenetic modifiers that regulate AIM2 are influenced by environmental cues, including cytokines IL‐21, IFNα and high salt, adding another layer of complexity. Considering targeting cell‐type specific factors and combinatorial therapies would bring better hopes to patients with this serious disease.

## CONFLICT OF INTEREST

The author declares that there is no potential conflict of interest.
